# Supplementary material for: Relationship between intended force and actual force: comparison between athletes and non-athletes
Source: PeerJ. 2024 Apr 3;12:e17156. doi: 10.7717/peerj.17156 (PMC10998631; doi:10.7717/peerj.17156)
Supplement: Supplemental Information 4 [file peerj-12-17156-s004.docx]

TABLE A. Accuracy (AC) non-statistically significant main effects and interactions. Repeated Measures Analysis of Variance (ANOVA).

|  | | | | | | | | | | | | | | |
| --- | --- | --- | --- | --- | --- | --- | --- | --- | --- | --- | --- | --- | --- | --- |
| **Cases** | | **Sphericity Correction** | | **Sum of Squares** | | **df** | | **Mean Square** | | **F** | | **p** | | **η² _p_** |
| Contractions ✻ Groups |  | None |  | 299.892 |  | 1.000 |  | 299.892 |  | 2.929 |  | 0.095 |  | 0.072 |
| Force intensities (% MVIC) ✻ Groups |  | None |  | 279.782 |  | 2.000 |  | 139.891 |  | 1.285 |  | 0.283 |  | 0.033 |
| Contractions ✻ Force intensities (% MVIC) ✻ Groups |  | None |  | 112.794 |  | 2.000 |  | 56.397 |  | 0.744 |  | 0.479 |  | 0.019 |
|  | | | | | | | | | | | | | | |

TABLE B. Aggregate Likert score non-statistically significant main effects and interactions. Repeated Measures Analysis of Variance (ANOVA).

|  | | | | | | | | | | | | | | | |
| --- | --- | --- | --- | --- | --- | --- | --- | --- | --- | --- | --- | --- | --- | --- | --- |
| **Cases** | | **Sphericity Correction** | | **Sum of Squares** | | **df** | | **Mean Square** | | **F** | | **p** | | **η² _p_** | |
| Contractions ✻ Groups |  | None |  | 0.037 |  | 1.000 |  | 0.037 |  | 0.009 |  | 0.925 |  | 2.368e -4 |  |
| Force intensities (% MVIC) |  | None |  | 14.908 |  | 2.000 |  | 7.454 |  | 1.800 |  | 0.172 |  | 0.045 |  |
| Contractions ✻ Force intensities (% MVIC) |  | None |  | 0.058 |  | 2.000 |  | 0.029 |  | 0.010 |  | 0.990 |  | 2.574e -4 |  |
| Contractions ✻ Force intensities (% MVIC) ✻ Groups |  | None |  | 0.025 |  | 2.000 |  | 0.013 |  | 0.004 |  | 0.996 |  | 1.103e -4 |  |
|  | | | | | | | | | | | | | | | |
